# *ASXL1* mutations with serum EPO levels predict poor response to darbepoetin alfa in lower-risk MDS: W-JHS MDS01 trial

**DOI:** 10.1007/s12185-022-03414-9

**Published:** 2022-07-12

**Authors:** Yasuyoshi Morita, Yasuhito Nannya, Motoshi Ichikawa, Hitoshi Hanamoto, Hirohiko Shibayama, Yoshinobu Maeda, Tomoko Hata, Toshihiro Miyamoto, Hiroshi Kawabata, Kazuto Takeuchi, Hiroko Tanaka, Junji Kishimoto, Satoru Miyano, Itaru Matsumura, Seishi Ogawa, Koichi Akashi, Yuzuru Kanakura, Kinuko Mitani

**Affiliations:** 1 Divison of Hematology and Rheumatology, Department of Internal Medicine, Kindai University Faculty of Medicine, Osaka-Sayama, Osaka Japan; 2 Department of Pathology and Tumor Biology, Kyoto University, Kyoto, Japan; 3 Division of Hematopoietic Disease Control, The Institute of Medical Science, The University of Tokyo, Tokyo, Japan; 4 Department of Hematology and Oncology, Dokkyo Medical University, 880, Kitakobayashi, Mibu-machi, Shimotsuga-gun, Tochigi, 321-0293 Japan; 5 Department of Hematology, Faculty of Medicine, Nara Hospital Kindai University, Nara, Japan; 6 Department of Hematology, National Hospital Organization Osaka National Hospital, Osaka, Japan; 7 Department of Hematology and Oncology, Okayama University Hospital, Okayama, Japan; 8 Department of Hematology, Atomic Bomb Disease Institute, Nagasaki University, Nagasaki, Japan; 9 Department of Medicine and Biosystemic Science, Kyushu University Graduate School of Medical Science, Fukuoka, Japan; 10 Department of Hematology, National Hospital Organization Kyoto Medical Center, Kyoto, Japan; 11 Department of Hematology, Clinical Immunology and Infectious Diseases, Ehime University Graduate School of Medicine, Ehime, Japan; 12 Human Genome Center, Institute of Medical Science, The University of Tokyo, Tokyo, Japan; 13 Center for Clinical and Translational Research, Kyushu University Hospital, Fukuoka, Japan; 14 M&D Data Science Center, Tokyo Medical and Dental University, Tokyo, Japan; 15 Institute for the Advanced Study of Human Biology (WPI-ASHBi), Department of Medicine, Kyoto University, Kyoto, Japan; 16 Center for Hematology and Regenerative Medicine, Karolinska Institute, Stockholm, Sweden; 17 Department of Hematology and Oncology, Graduate School of Medicine, Osaka University, Osaka, Japan; 18 Sumitomo Hospital, Osaka, Japan

**Keywords:** Darbepoetin alfa, *ASXL1* gene mutations, Erythropoietin, Anemia, Myelodysplastic syndromes

## Abstract

Darbepoetin alfa (DA) is used to treat anemia in lower-risk (IPSS low or int-1) myelodysplastic syndromes (MDS). However, whether mutations can predict the effectiveness of DA has not been examined. The present study aimed to determine predictive gene mutations. The primary endpoint was a correlation between the presence of highly frequent (≥ 10%) mutations and hematological improvement-erythroid according to IWG criteria 2006 by DA (240 μg/week) until week 16. The study included 79 patients (age 29–90, median 77.0 years; 52 [65.8%] male). Frequently (≥ 10%) mutated genes were *SF3B1* (24 cases, 30.4%), *TET2* (20, 25.3%), *SRSF2* (10, 12.7%), *ASXL1* (9, 11.4%), and *DNMT3A* (8, 10.1%). Overall response rate to DA was 70.9%. Multivariable analysis including baseline erythropoietin levels and red blood cell transfusion volumes as variables revealed that erythropoietin levels and mutations of *ASXL1* gene were significantly associated with worse response (odds ratio 0.146, 95% confidence interval 0.042–0.503; *p* = 0.0023, odds ratio 0.175, 95% confidence interval 0.033–0.928; *p* = 0.0406, respectively). This study indicated that anemic patients who have higher erythropoietin levels and harbor *ASXL1* gene mutations may respond poorly to DA. Alternative strategies are needed for the treatment of anemia in this population. Trial registration number and date of registration: UMIN000022185 and 09/05/2016.

## Introduction

Myelodysplastic syndromes (MDS) are clonal stem cell disorders characterized by ineffective hematopoiesis, and occasionally progress to acute myelogenous leukemia (AML) [1, 2]. The pathogenesis of MDS is thought to be a multistep process involving two or more genetic alterations that cause clonal proliferation of an abnormal stem cell [3, 4]. Our understanding of the molecular pathogenesis of MDS has improved in recent years, mainly through the identification of major mutational targets [5–9]. The majority of patients with lower-risk MDS of International Prognostic Scoring System (IPSS) [10] low or intermediate-1 risk have symptoms of anemia due to ineffective erythropoiesis [11], and need therapeutic intervention, including red blood cell (RBC) transfusion. Although RBC transfusions can temporarily reduce anemic symptoms, frequent transfusions usually lead to iron overload, which is associated with reduced survival and lower quality of life [12].

On the other hand, the development of erythropoiesis in the fetal liver and adult bone marrow is regulated by the hormone erythropoietin (EPO) [13]. Therefore, recombinant human EPO (rHuEPO) and other erythropoiesis-stimulating agents (ESAs) are used for the treatment of MDS-related anemia [14]. In Japan, darbepoetin alfa (DA), which is a re-engineered form of EPO, has been approved for clinical practice. Around 50% of the patients respond to EPO ± granulocyte-colony stimulating factors (G-CSF), and the median duration of response is 2 years [15, 16]. DA has also been reported to show an overall response rate of about 60% when employed for the treatment of anemia in lower-risk MDS [17].

Prediction of response to ESAs in anemic MDS patients is often based on clinical biomarkers such as volume of RBC transfusion, serum EPO levels, ferritin, and IPSS/IPSS-R [18], and the use of Nordic score combined with serum EPO levels and transfusion volume has also been proposed [19]. However, the relationship between molecular pathogenesis of MDS and responsiveness to DA has not yet been well studied. Here we report the results of the West Japan Hematology Study Group (W-JHS) MDS01 trial, to determine gene mutations that predict the effectiveness of DA in treating anemia of lower-risk (low or int-1 in IPSS [10] risk category) MDS.

## Materials and methods

### Patient eligibility

Patients with lower-risk MDS (low or int-1 in IPSS [10] category) were registered in the W-JHS MDS01 study (UMIN000022185) between February 2016 and May 2019 at 36 institutions in Japan. Other eligibility criteria were as follows: newly diagnosed patients with definite MDS based on diagnostic criteria of FAB classification [20] and the 4th edition of World Health Organization (WHO) classification [21]; patients having anemia associated with MDS, clinically eligible for DA treatment and of age 16 years or older. Patients with present or past medical record of myocardial infarction, pulmonary infarction, cerebral infarction or similar disorders or risk of thromboembolism, uncontrollable hypertension, prior treatment with DA or other formulations of EPO, severe (requiring hospital care or judged by investigators) or uncontrollable complication, and those judged inappropriate for study participation due to complication of mental disease or psychiatric symptom and cognitive disorder were excluded. This study is registered at the University Hospital Medical Information Network Clinical Trials Registry on 09/05/2016, with ID No. 000022185.

### Procedure

DA at a dose of 240 µg per body was administered once weekly for 16 weeks. Analysis was performed to confirm whether the presence of a specific gene mutation with a frequency of ≥ 10% affects the efficacy of DA. Peripheral blood sample of the subjects was collected before administration of DA, and genomic DNA was extracted. The presence of gene mutations was then analyzed with a panel of 376 genes in a previous report [6] using a next-generation sequencing method. In brief, 376 known target genes in MDS were examined for mutations in 79 patients from the cohort, using massively parallel sequencing (Illumina, Inc., San Diego, CA, USA) of SureSelect (Agilent Technologies Inc., Santa Clara, CA, USA)-captured target sequences. All sequencing data were analyzed using our in-house pipeline [22], through which highly probable oncogenic mutations were called by eliminating sequencing/mapping errors using an empirical Bayesian approach [23] and known/possible SNPs based on the available databases.

### Assessment of response

The primary endpoint was a correlation between highly frequent (≥ 10%) gene mutations and hematological improvement-erythroid (HI-E) according to the International Working Group (IWG) criteria 2006 [24] at week 16 after the initiation of DA treatment. Secondary endpoints were major and minor responses to DA at week 16 after the initiation of treatment in blood transfusion-dependent subjects, HI-E in blood transfusion-independent and dependent subjects, variety and frequency of gene mutations observed in all subjects, the correlation between highly frequent gene mutations and interval to achievement of the first HI-E according to the IWG criteria 2006 [24], and mortality (overall survival, OS) and progression to AML (progression-free survival, PFS) from 16 weeks to 1 year after the initiation of treatment.

The IWG criteria 2006 (HI-E) were used, defined as either hemoglobin increased by 1.5 g/dL or more compared to pre-treatment (< 11.0 g/dL) or RBC transfusion volume/8 weeks decreased by more than 4 units in RBC transfusion-dependent subjects. In Japan, one unit of red blood cell preparation is produced from 200 ml of whole blood. As for transfusion-dependent subjects, the major response was defined as no need for RBC transfusion (withdrawal from RBC transfusion dependence) for more than 56 consecutive days, and increase in the highest Hb concentration during the withdrawal period by at least 1.0 g/dL compared to the baseline Hb concentration, while the minor response was defined as ≥ 50% decrease in RBC transfusion volume over 56 consecutive days compared to baseline transfusion volume. For AML progression, progression to AML or death without progression were stated as events, and the subjects without confirmed progression to AML were censored at the date of the last known survival. For overall survival (OS), death from any cause was considered an event, and for the survival cases, the study was terminated at the date of the last known survival.

### Assessment of safety

All adverse events (AEs) were recorded in subjects who received DA at least once from the first administration to day 29 of the last cycle, and classified according to the Common Terminology Criteria for Adverse Events Version 4.0 [25]. For the subjects who dropped out before completion of the study, AEs were monitored for two weeks after the last administration.

### Statistical analysis

Statistical significance of EPO levels before the treatment between non-responders and responders was evaluated by the Wilcoxon rank-sum test. Correlation between numbers of gene mutations and response to DA was analyzed by the Cochran-Armitage trend test and the chi-squared test.

Odds ratios between the gene mutations and the outcomes were estimated using univariate and multivariable logistic regression models. The multivariable analysis was adjusted for baseline EPO levels (low: < 100, high: ≥ 100 mIU/mL) and RBC transfusion volumes (low: < 1 unit, high: ≥ 1 unit/month) as the explanatory variables.

The survival curves were estimated using Kaplan–Meier methods, and the confidence intervals for the median survival time (MST) and the annual survival rate were calculated using Brookmeyer and Crowley's method and Greenwood's formula, respectively. In all analyses, *p* < 0.05 (two-sided) was considered statistically significant. Statistical analysis was performed using SAS Ver. 9.4.

### Ethics

This study was conducted in compliance with the Act on the Protection of Personal Information (Act No. 57 of May 30, 2003), the Declaration of Helsinki (October 2013, translated by the Japanese Medical Association in the revised version of Fortaleza), the Clinical Research Act (Act No. 16 of 2017), the Ordinance for Enforcement of the Clinical Research Act (Ordinance No. 17 of the Ministry of Health, Labour and Welfare of 2018), and the Ethical Guidelines for Human Genome/Gene Analysis Research (February 28, 2017).

The protocol and an explanatory document regarding the protocol provided to patients were approved by the Ethics Review Committee of each participating institution. Prior to subject enrollment, the content of the study was explained to the patients using the explanatory document, and written informed consent was obtained from all participants. If a patient was under 20 years of age, written informed consent of the patient and his or her guardian was obtained.

## Results

### Subjects

A total of 85 patients underwent enrollment screening. Of these, 79 subjects were included in the full analysis set (FAS), after excluding 4 ineligible patients and 2 patients who withdrew their consent before the start of protocol treatment. The median (range) follow-up for FAS was 374 (44–1094) days. Baseline characteristics of the 79 subjects are shown in Table 1. Median (range) age was 77.0 (29–90) years; 52 males (65.8%) and 27 females (34.2%) were included in the study. Median (range) Hb level was 8.1 g/dL (4.3–11.8 g/dL) in FAS, and 7.8 g/dL (4.3–10.2 g/dL) and 8.1 g/dL (4.9–11.8 g/dL) in transfusion-dependent and non-transfusion-dependent subjects, respectively. The number of transfusion-dependent cases was 15 (19.0%).

**Table 1:** Baseline characteristics of the subjects

Males/females, *n* (%)	52/27 (65.8/34.2)
Median age, years (range)	77.0 (29–90)
Median hemoglobin (g/dL)	8.1 (4.3–11.8)
Transfusion-dependent cases	7.8 (4.3–10.2)
Non-transfusion-dependent cases	8.1 (4.9–11.8)
WHO classification (the 4th edition), *n* (%)	
RCUD	23 (29.1)
RARS	12 (15.2)
RCMD	37 (46.8)
RAEB-1	5 (6.3)
RAEB-2	0 (0.0)
MDS-U	1 (1.3)
MDS with isolated del (5q)	1 (1.3)
WHO classification (the revised 4th edition), *n* (%)	
MDS-SLD	21 (26.6)
MDS-MLD	31 (39.2)
MDS-RS-SLD	14 (17.7)
MDS-RS-MLD	6 (7.6)
MDS with isolated del(5q)	1 (1.3)
MDS-EB-1	5 (6.3)
MDS-EB-2	0 (0.0)
Unknown	1 (1.3)
IPSS, *n* (%)	
Low	29 (36.7)
Int-1	50 (63.3)
Int-2	0 (0.0)
High	0 (0.0)
IPSS-R, *n* (%)	
Very low	6 (7.6)
Low	42 (53.2)
Intermediate	28 (35.4)
High	2 (2.5)
Very high	1 (1.3)
Transfusion dependency, *n* (%)	
No	64 (81.0)
Yes	15 (19.0)
EPO, *n* (%)	
< 100	40 (50.6)
≧ 100	39 (49.4)

*WHO* World Health Organization, *RCUD* refractory cytopenia with unilineage dysplasia, *RARS* refractory anemia with ring sideroblasts, *RCMD* refractory cytopenia with multilineage dysplasia, *RAEB-1* refractory anemia with excess blasts-1, *RAEB-2* refractory anemia with excess blasts-2, *MDS-U* myelodysplastic syndromes-unclassified, *MDS-SLD* myelodysplastic syndromes with single lineage dysplasia, *MDS-MLD* MDS with multilineage lineage dysplasia, *MDS-RS* with ring sideroblasts, *MDS-EB MDS* with excess blasts, *IPSS* International Prognostic Scoring System, *IPSS-R* revised IPSS, *EPO* erythropoietin, *FAS* full analysis set

### Outcomes

**Fig. 1:**
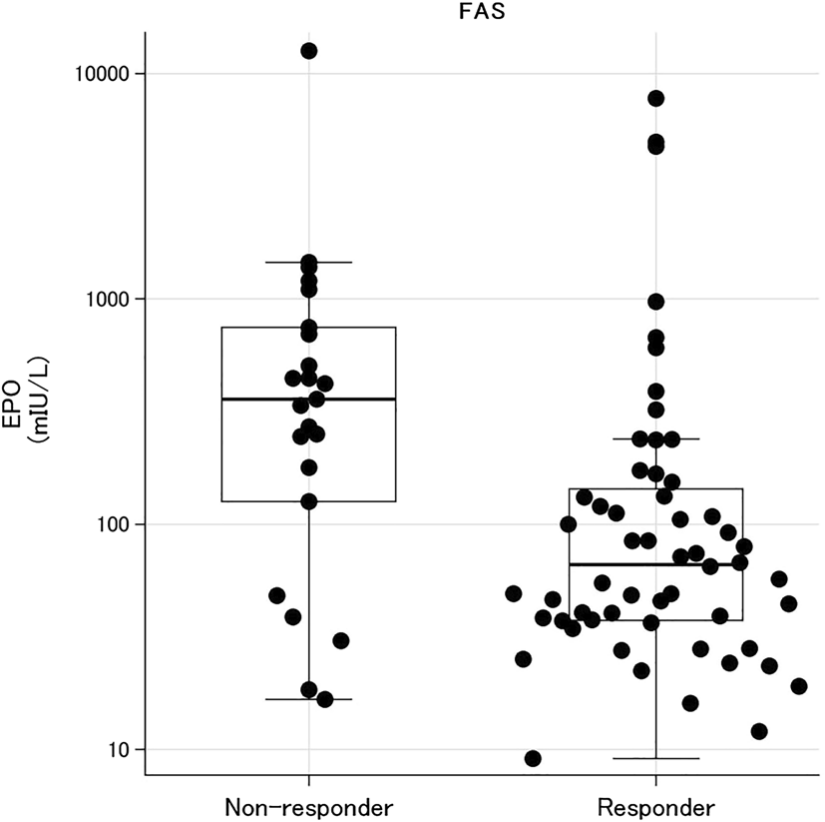
EPO levels before the DA treatment in the non-responders and responders. The beeswarm boxplot of EPO levels in non-responders (*N* = 23) and responders (*N* = 56) by the IWG criteria 2006 are shown with median and the first and third quartile levels, indicating a statistical difference between them (*p* = 0.0008)

Rate of overall response (achievement of HI-E according to the IWG criteria 2006) was 70.9% (60.0% in transfusion-dependent cases, and 73.4% in non-transfusion-dependent cases) (Table 2). Major/minor responses were observed in 46.7% and 60.0% of the RBC transfusion-dependent subjects (*n* = 15). When compared levels of EPO before the treatment between non-responders (*n* = 23) and responders (*n* = 56) by the IWG criteria 2006, the levels were significantly lower in responders than non-responders (*p* = 0.008) (Fig. 1). The median (range) levels of non-responders and responders were 358.0 (16.7–1450.0) mIU and 66.3 (9.1–239.0) mIU/L, respectively.

**Table 2:** Rate of overall response to DA at week 16

	*N* = 79	HI-E (%)	95% CI	
			Lower	Upper
IWG criteria				
All subjects	79	70.9	60.1	79.7
Transfusion independent	64	73.4	61.5	82.7
Transfusion dependent	15	60.0	35.7	80.2
Major response				
Transfusion dependent	15	46.7	24.8	69.9
Minor response				
Transfusion dependent	15	60.0	35.7	80.2

*HI-E* hematological improvement-erythroid, *DA* darbepoetin alfa, *IWG* International Working Group

**Fig. 2:**
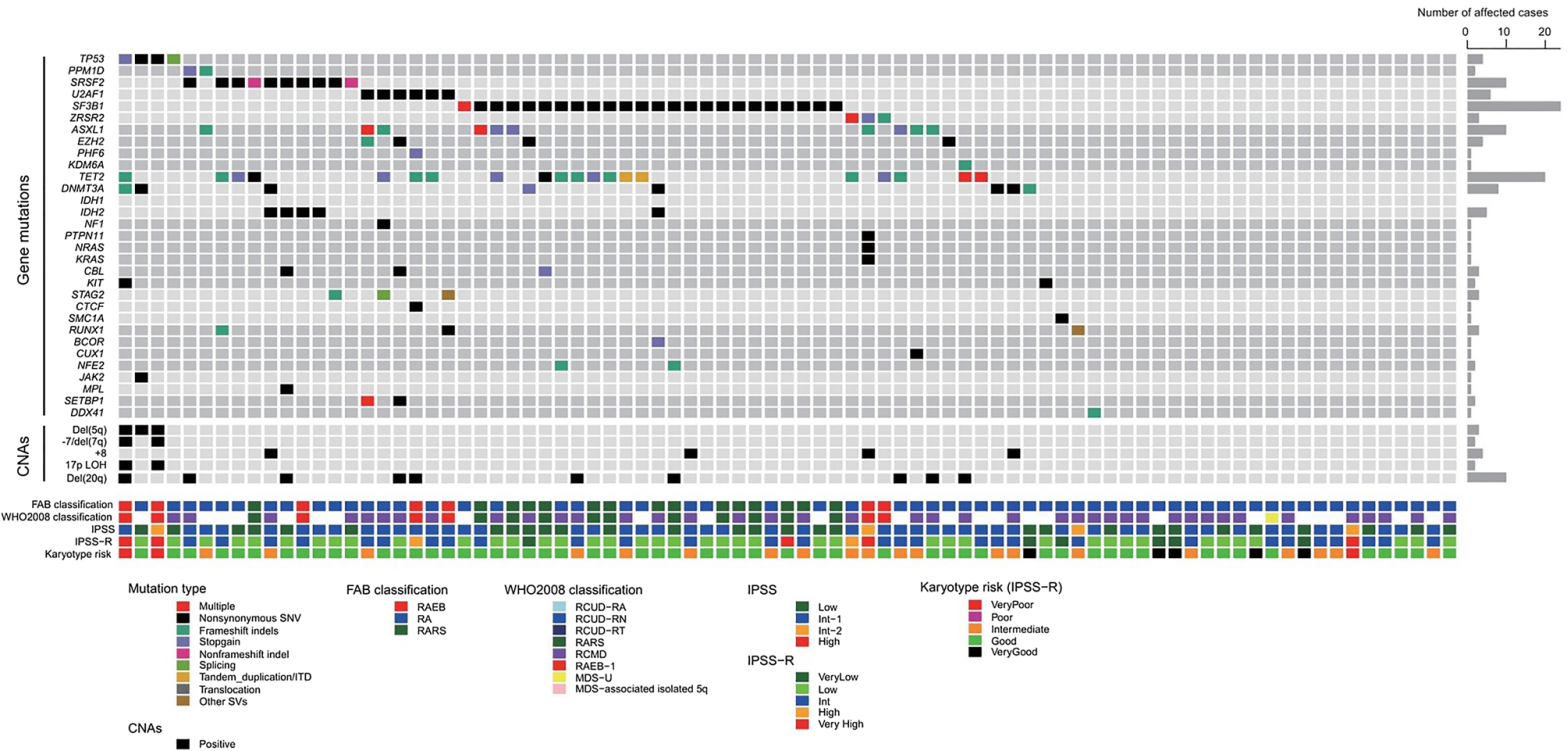
Landscape of genetic alterations in the 83 cases. The genetic alterations in the 83 cases from this study cohort are shown. Disease classifications (FAB- and WHO 2008-based), disease risk (IPSS- and IPSS-R-based), and karyotype risks (IPSS-R karyotype risk) together with the number of affected cases are shown by color as indicated. *SNV* single nucleotide variant, *ITD* internal tandem duplication, *CNA* copy number alterations

We analyzed 376 genes for mutations on 79 samples. The results are shown in Fig. 2. The highly frequent (10% or more) gene mutations included those in *SF3B1* (24 cases, 30.4%), *TET2* (20 cases, 25.3%), *SRSF2 (*10 cases, 12.7%), *ASXL1* (9 cases, 11.4%), and *DNMT3A* (8 cases, 10.1%). As reported previously, RNA splicing- and epigenetics-regulating genes were major targets for mutations in this study.

At first, we studied the rate of HI-E for each number of gene mutations. The rates were 63.9% (23 cases/36 cases), 88.9% (8/9), 75.0% (9/12), 66.7% (6/9), 87.5% (7/8), 50.0% (1/2), 100.0% (1/1), 100.0% (1/1) and 0% (0/1) in 0, 1, 2, 3, 4, 5, 6, 7 and 8 mutations, respectively. The Cochran-Armitage trend test indicated that there was no correlation between numbers of gene mutations and response to DA (*p* = 0.7084). When compared the response between the presence or absence of gene mutations, and, the presence of > 2 or ≤ 2 gene mutations, we still could not notice any differences (*p* = 0.210 and 0.823, respectively).

We next evaluated the relationship between the presence of highly frequent (10% or more) mutated genes and the rate of HI-E by univariate analysis in FAS (Table 3). The univariate logistic regression analysis showed no significant association between these mutations with a frequency of ≥ 10% and therapeutic efficacy of DA. The same results were obtained when the analysis was limited to RBC non-transfusion-dependent subjects (*n* = 64).

**Table 3:** Association between gene mutation and response to DA at week 16: univariate analysis

Gene	*N* (%)	Odds ratio	95% CI		*p* value
*SF3B1*	24 (30.4%)	2.639	0.788	8.839	0.116
*TET2*	20 (25.3%)	2.906	0.761	11.104	0.119
*SRSF2*	10 (12.7%)	1.750	0.342	8.951	0.502
*ASXL1*	9 (11.4%)	0.277	0.067	1.145	0.076
*DNMT3A*	8 (10.1%)	1.260	0.235	6.757	0.787

*DA* darbepoetin alfa

In the multivariable analysis including baseline EPO levels and RBC transfusion volumes as variables, mutation of *ASXL1* gene as well as baseline EPO levels was identified to independently predict poor response to DA with statistical significance (odds ratio 0.180, 95% CI 0.035–0.928, *p* = 0.040 for *ASXL1* mutation, odds ratio 0.146, 95% confidence interval 0.042–0.503; *p* = 0.0023 for EPO levels) (Table 4). Transfusion volumes were not detected as predictive factors, possibly because that major part of our cohort was transfusion-independent. Response rates in subjects with low EPO (< 100 mIU/mL) + *ASXL1* mutation(−) (*n* = 35), low EPO + *ASXL1* mutation(+) (*n* = 5), high EPO (≥ 100 mIU/mL) + *ASXL1* mutation(−), (*n* = 35) and high EPO + *ASXL1* mutation(+) (*n* = 4) were 88.6, 80.0, 60.0 and 0%, respectively (Table 5). The result of chi-squared test showed that the four groups were significantly different in terms of response (*p* = 0.0006).

**Table 4:** Association between achievement of HI-E and gene mutation combined with EPO level and transfusion volume: multivariable analysis

Variable	Odds ratio	95% CI		*p* value
SF3B1 (mutation(+)/mutation(−))	2.776	0.767	10.048	0.1197
Baseline EPO (mIU/mL) (100+/< 100)	0.170	0.053	0.546	0.0029
Baseline TF (unit/month) (1+/< 1)	0.797	0.257	2.468	0.6936
TET2 (mutation(+)/mutation(−))	2.361	0.573	9.732	0.2344
Baseline EPO (mIU/mL) (100+/< 100)	0.188	0.059	0.602	0.0049
Baseline TF (unit/month) (1+/< 1)	0.799	0.259	2.465	0.6959
SRSF2 (mutation(+)/mutation(−))	1.226	0.212	7.099	0.8202
Baseline EPO (mIU/mL) (100+/< 100)	0.177	0.055	0.563	0.0034
Baseline TF (unit/month) (1+/< 1)	0.818	0.269	2.491	0.7237
**ASXL1 (mutation(+)/mutation(−))**	**0.175**	**0.033**	**0.928**	**0.0406**
Baseline EPO (mIU/mL) (100+/< 100)	0.146	0.042	0.503	0.0023
Baseline TF (unit/month) (1+/< 1)	0.766	0.238	2.460	0.6537
DNMT3A (mutation (+)/mutation(−))	1.093	0.166	7.193	0.9262
Baseline EPO (mIU/mL) (100+/< 100)	0.175	0.055	0.554	0.0031
Baseline TF (unit/month) (1+/< 1)	0.808	0.258	2.535	0.7154

*HI-E* hematological improvement-erythroid, *EPO* erythropoietin, *TF* transfusion, *CI* confidence interval

**Table 5:** Responsive rates to DA in the subgroup classified by baseline EPO levels and the presence or absence of *ASXL1* mutations

Subgroup (*N*)	*ASXL1* mutations (−)	*ASXL1* mutations (+)
Lower EPO level (< 100 mIU/mL)	88.6% (35)	80.0% (5)
Higher EPO level (≥ 100 mIU/mL)	60.0% (35)	0% (4)

*EPO* erythropoietin

### Correlation between highly frequent gene mutations and interval to the achievement of the first HI-E

Of the 79 subjects who were included in FAS, 56 subjects achieved the HI-E according to the IWG criteria 2006, and the median time to achievement (95% CI) was 7.1 weeks (6.1–10.1 weeks). After adjustment for the baseline EPO levels (cut-off, 100 mIU/mL), although none of the highly frequent gene mutations had a significant association with the time to achievement of the first HI-E according to the IWG criteria 2006, mutation of *ASXL1* gene showed a tendency of later achievement: (MST of mutation(+)/mutation(−), not reached/6.7 weeks, *p* = 0.1649).

### PFS and OS

Progression to AML was observed in 24 subjects from week 0 to year 1, and in 10 subjects from week 16 to year 1. A total of 23 subjects died between week 0 and year 1, and 9 subjects died between week 16 and year 1.

**Fig. 3:**
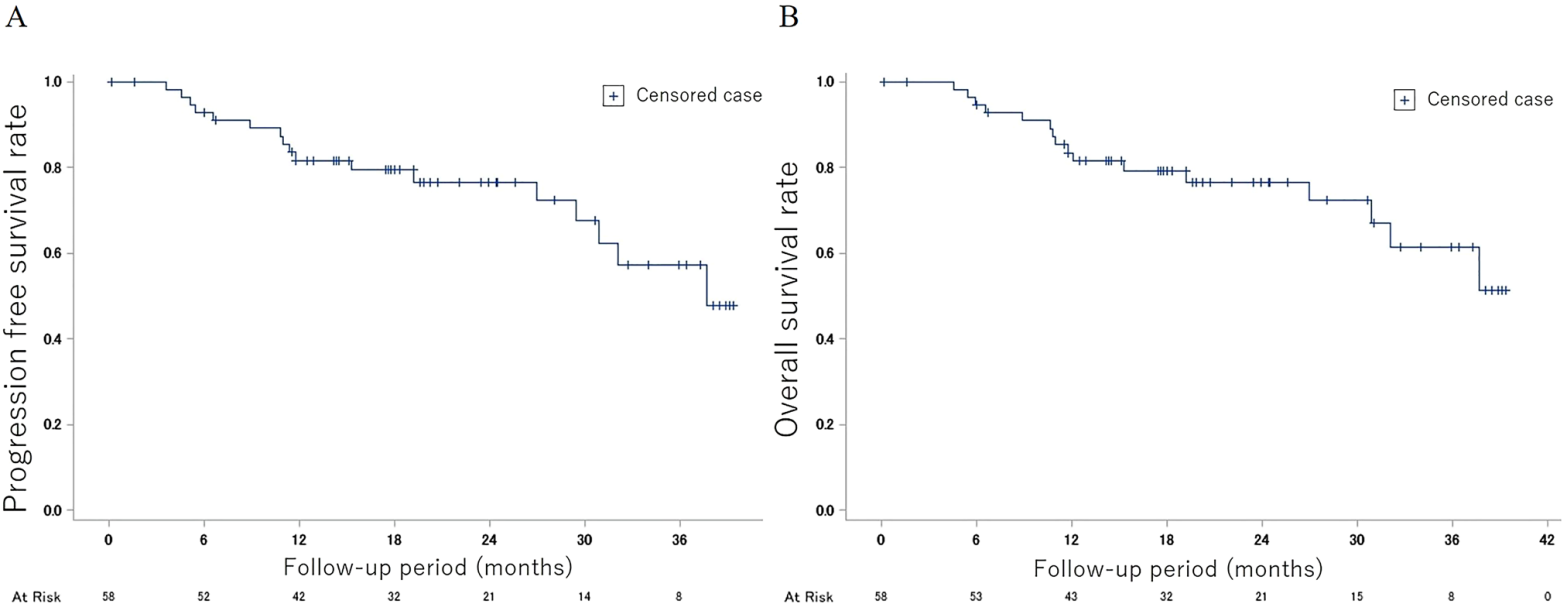
**A** Progression-free survival to AML. Data in 58 subjects who continued treatment without progression to acute myelogenous leukemia till week 16. Initial date of reckoning: 16 weeks after the start of treatment. **B** Overall survival. Data in 58 subjects who continued treatment without progression to acute myelogenous leukemia till week 16. Initial date of reckoning: 16 weeks after the start of treatment

PFS and OS at from week 16 to year 1 after the initiation of treatment are shown on the 58 subjects who continued treatment without progression to AML until week 16 in Fig. 3 A and B. PFS (95% CI) at year 1 was 81.7% (68.6–89.7%) with MST (95% CI) of 37.7 months (29.5 months–not reached), and OS at year 1 was 83.5% (70.7–91.1%) with MST (95% CI) not reached (30.9 months–not reached).

### Safety

Grade 3/4 adverse events observed during the study period were anemia in 33 subjects (41.8%), neutrophil count decreased in 24 (30.4%), platelet count decreased in 18 (23.4%), white blood cell count decreased in 16 (20.3%), lymphocyte count decreased in 15 (19.0%), hyperglycemia in 3 (5.9%), hypoalbuminemia in 2 (3.1%), and aspartate aminotransferase increased in 1 (1.3%).

## Discussion

In this study focusing on DA-eligible lower-risk MDS patients, gene mutations frequently observed in MDS were found in *SF3B1* (30.4%), *TET2* (25.3%), *SRSF2* (12.7%), *ASXL1* (11.4%), and *DNMT3A* (10.1%), which is consistent with a previous report [6]. The response rate of DA up to 16 weeks was 70.9% based on the IWG criteria (60.0% in transfusion-dependent cases, and 73.4% in non-transfusion-dependent cases). EPO levels before the treatment were significantly lower in the responders than non-responders. Regarding the association between gene mutations at a frequency of 10% or more and the response rate of DA, the univariate analysis showed no significant association between them. Multivariable analysis that included serum EPO levels and RBC transfusion volumes revealed that the presence of *ASXL1* gene mutations and higher EPO levels (≥ 100 mIU/mL) were independent factor that predicts for poor response to DA. In a previous meta-analysis, it had been shown that the serum EPO level < 100 IU/L is a biomarker for the effectiveness of DA [26]. Similarly, in our study, DA therapy was effective in subjects with low EPO levels (< 100 mIU/mL), irrespective of the absence (response rate, 88.6%) or presence (response rate, 80.0%) of *ASXL1* mutations. However, it is interesting to note that about 60% of the subjects with serum EPO levels ≥ 100mIU/mL and without *ASXL1* mutations responded to DA therapy in our cohort. Even in patients with higher levels of EPO, there may be a chance to recover from anemia by DA therapy if they do not possess *ASXL1* mutations. One-year PFS and OS rates was 81.7% (95% CI 68.6–89.7%) and 83.5% (70.7–91.1%), respectively. Considering that our cohort included 5 subjects with RAEB-1 according to the WHO classification, the PFS and OS were not worse than expected.

DA is an ESA in which sustainable serum concentration has been obtained by substituting 5 amino acids in EPO [27, 28]. Like EPO, DA binds to erythropoietin receptors and thereby promotes erythropoiesis in early and late erythroid progenitor cells in the bone marrow [27, 29]. When injected subcutaneously once a week, sufficient serum levels are maintained for the treatment of anemia [28]. Nine studies performed in patients with MDS reported response rates to DA according to the IWG 2000 criteria with a range of 38–72.5% within 12–24 weeks [26]. Similarly, in the present study, overall response rate to DA at week 16 was 70.9% according to the IWG criteria 2006. There was a tendency that non-transfusion-dependent subjects had a better response to DA than transfusion-dependent subjects, with ORR of 73.4 and 60.0%, respectively. The earlier commencement of DA could enable avoidance of transfusion dependency in anemic patients with lower-risk MDS.

Kosmider et al*.* have reported that having > 2 somatic mutations was associated with lower HI-E in the ESA treatments for lower-risk MDS [30]. This result was opposite to ours. In our study, there was no co-relation between numbers of gene mutations and response to DA (*p* = 0.7084). Even though we divided patients into two groups by the mutation numbers of > 2 or ≤ 2 like in their study, no differences in the response were observed (*p* = 0.823). Although the sample size was the same (*n* = 79) and response rates were similar (70.9 and 64.5% according to the IWG 2006 criteria in our and their studies), these two studies had quite different designs. Our study was prospective, while Kosmider et al.’s study retrospective. Patients in our cohort were consistently treated with DA at a dose of 240 μg/week, while those in their cohort with EPO or DA at various doses with or without G-CSF. We analyzed 326 genes for mutations, but they only 37 genes. All these may have caused different conclusions.

They also claimed that individual mutations of the frequently mutated genes had no significant impact on HI-E by the univariate analysis, which was the same result as ours. Importantly, however, by using multivariable analysis that included serum EPO level and RBC transfusion volumes as variables, we showed that the presence of *ASXL1* mutations besides EPO level was significantly associated with poor response to DA among the highly frequent (> 10%) gene mutations observed in this study such as those in *SF3B1, TET2, SRSF2, ASXL1,* and *DNMT3A*. Because MDS are heterogenous not only in morphology but also in molecular pathogenesis, it was difficult for less frequent gene mutations to evaluate their predictive value on the DA treatment. Another important result of this study was that *ASXL1* gene mutations were also correlated with possible prolonged interval to the achievement of the first HI-E. *ASXL1* mutations are known genetic factors that predict unfavorable clinical courses [31], with a high rate of progression to AML [32]. Considering a balance between cost and benefit, DA perhaps should not be applied to patients with lower-risk MDS showing a higher level of EPO and carrying *ASXL1* mutations.

*ASXL1* is an epigenetics-regulating gene that supports the functions of polycomb complex PRC1 and represses the expression of oncogenes and other genes through methylation of K4 in histone H3 [33]. It has also been shown that mutant *ASXL1* disrupts the function of PRC1 and causes derepression of expression in target genes [34], which possibly leads to the development of myeloid malignancies, including MDS [35]. The candidate target genes for the derepression include *HOXA9* and *MIR125A* [36]*.* While *HOXA9* is a known oncogene in hematopoietic tumors of the myeloid lineage [37], *MIR125A* is suggested to impair hematopoietic cell differentiation [38]. On the other hand, Shi et al. reported that *ASXL1* loss impairs erythroid development and hinders erythroid differentiation [39], indicating that ineffective erythropoiesis of MDS may occur as a result of *ASXL1* mutation. Although the precise mechanism in a regard to poor response to DA could not be identified for *ASXL1*-mutated subjects, the mutation could induce refractoriness to DA. From another point of view, Raimbault et al. reported that the low expressions of CD117/c-KIT^+^ in lower-risk MDS erythroid precursors was correlated with ESA failure [40]. It would be interesting to analyze the association between *ASXL1* gene mutations and expression levels of CD117/c-KIT^+^ in a future study.

This prospective study provided the first evidence in the ESA therapy that the existence of some specific gene mutations (*ASXL1* mutations) may be associated with response to specific treatments (DA). In our opinion, even though patients with lower-risk MDS show higher EPO levels, DA would be effective if they do not carry *ASXL1* mutations. However, when patients have both the predictive factors of poor response, namely, higher levels of EPO and *ASXL1* mutations, alternative therapies would be recommended as the first-line therapy for anemia. One of such candidates may be luspatercept which is a recombinant fusion protein binding TGF-β superfamily ligands and is effective in patients with increased ring sideroblasts and/or *SF3B1* mutations [41]. Even in cytokine and other supportive therapies for anemia, molecular stratification needs to be established to determine their application in the near future.
